# Differences in hip morphology between the sexes in patients undergoing hip resurfacing

**DOI:** 10.1186/1749-799X-5-76

**Published:** 2010-10-15

**Authors:** Henry D Atkinson, Karanjeev S Johal, Charles Willis-Owen, Steven Zadow, Roger D Oakeshott

**Affiliations:** 1 Department of Trauma and Orthopaedics and North London Sports Orthopaedics, North Middlesex University Hospital, Sterling Way, London N18 1QX, UK; 2 Sportsmed SA, 32 Payneham Road, Stepney 5069, Adelaide, South Australia, Australia; 3 St Andrews Hospital Radiology Department, 350 South Terrace, Adelaide SA 5000, Australia

## Abstract

There is limited morphological data on the sex differences between the commonly used pelvic parameters. This study analysed the CT scans of 100 consecutive Caucasian patients, 61 males and 39 females, undergoing hip resurfacing arthroplasty surgery for hip osteoarthritis in one institution.

There were no sex differences in femoral torsion/anteversion, femoral neck angle and acetabular inclination. Males had a mean femoral torsion/anteversion of 8 degrees (range -5 to 26 degrees), a mean femoral neck angle of 129 degrees (range 119 to 138 degrees) and a mean acetabular inclination of 55 degrees (range 40 to 86 degrees). Females had a mean femoral torsion/anteversion of 9 degrees (range -2 to 31 degrees), a mean femoral neck angle of 128 degrees (range 121 to 138) and a mean acetabular inclination of 57 degrees (range 44 to 80 degrees). Females had a significantly greater acetabular version of 23 degrees (range 10 to 53) compared with 18 degrees in males (range 7 to 46 degrees (p = 0.02) and males had a significantly greater femoral offset of 55 mm (range 42 to 68 mm) compared with 48 mm (range 37 to 57 mm) in females (p = 0.00). There were no significant differences between measurements taken from each patient's right and left hips.

These findings may be useful for the future design and the implantation of hip arthroplasty components.

## Introduction

Metal on metal hip resurfacing is a modality of treatment that been shown to be an effective medium term solution for young adults with hip osteoarthritis[1]. Its principle is to preserve femoral bone stock and thus make any future revision surgery more amenable [2].

Accurate placement of the hip resurfacing components has an impact on the clinical outcomes. In particular, varus angulation of the femoral component and femoral neck notching have been shown to be risk factors for neck of femur fracture [3], and inadequate cup anteversion has a significant impact on hip flexion and edge loading [4].

Pre-operative calculations of femoral torsion, version and offset as well as acetabular version and inclination have been previously undertaken using plain radiographs [5,6], and subsequently more accurately using CT scanning [7]. The use of CT guidance in the placement of prostheses has been shown to significantly improve the post-operative component positions compared with freehand techniques [8]. CT scanning may also identify other anatomical anomalies and the presence of femoral head/neck cysts that may not be apparent on plain radiography [9].

Differences between male and female pelvic morphology may also be an important factor. Studies in childhood have demonstrated no difference in femoral anteversion between the sexes [10], however there may be differences in adults [11-17]. There continues to be very limited data on the sex differences between the commonly used pelvic parameters.

This study prospectively collected data from the CT scans of 100 consecutive Caucasian patients undergoing hip resurfacing for early osteoarthritis, analysing each of the patient's two hips for femoral neck angle, torsion and offset, and acetabular inclination and anteversion, and comparing the sexes. This, to our knowledge, is the first study of its kind.

## Patients and Methods

100 consecutive Caucasian patients, 61 males and 39 females (mean age 52 and 54 respectively), underwent pelvic CT scanning as part of their routine work-up for hip resurfacing arthroplasty surgery for hip osteoarthritis, between March 2007 and October 2008 in one institution.

The radiographic measurements were performed by two independent investigators. Repeated measurements were also taken in a random order to check for intraobserver error. The radiographic parameters measured included the femoral neck angle, femoral torsion, femoral offset, acetabular version and acetabular inclination.

**Figure 1:**
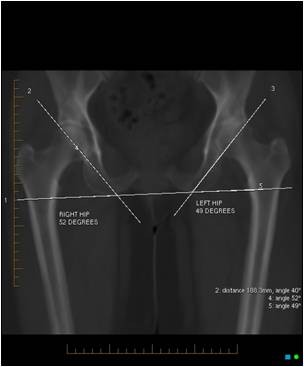
**The acetabular inclination calculated by plotting a trans-ischial line, and intersecting this with a second line drawn across the superior and inferior acetabular rims**.

The acetabular inclination was calculated by plotting a trans-ischial line on the pilot image, and a second line drawn across the superior and inferior rims, across the face of the bony acetabulum [18]. The inclination angle was formed by the intersection of these lines (Figure 1).

**Figure 2:**
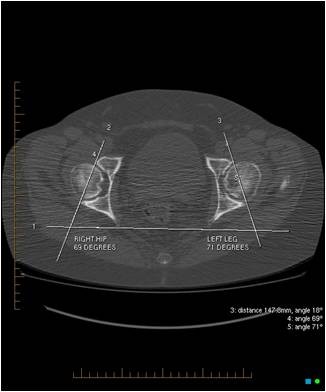
**The acetabular version calculated by plotting a trans-ischial line across the ischial tuberosities on axial CT image**. A second line is drawn across the anterior and posterior margins of the bony acetabulum. The anteversion angle is formed by the intersection of these two lines.

The acetabular version was calculated by plotting a trans-ischial line across the ischial tuberosities on the axial CT image. The trans-ischial line was then transposed to an axial image of the acetabulum, and a second line was drawn across the anterior and posterior margins of the bony acetabulum. The anteversion angle was formed by the intersection of these two lines (Figure 2).

**Figure 3:**
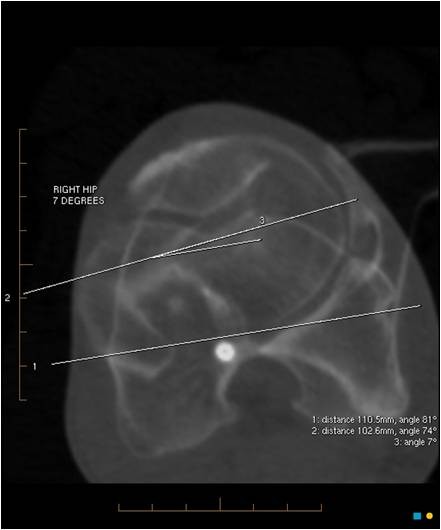
**The femoral torsion is determined by plotting a reference line through the transcondylar plane of the distal femur, and superimposing a second line through the axis of the neck of the femur**.

The femoral torsion was determined by plotting a reference line through the transcondylar plane of the distal femur, and plotting a second line through the axis of the neck of the femur. Superimposition of these lines gave the femoral torsion angle (Figure 3).

**Figure 4:**
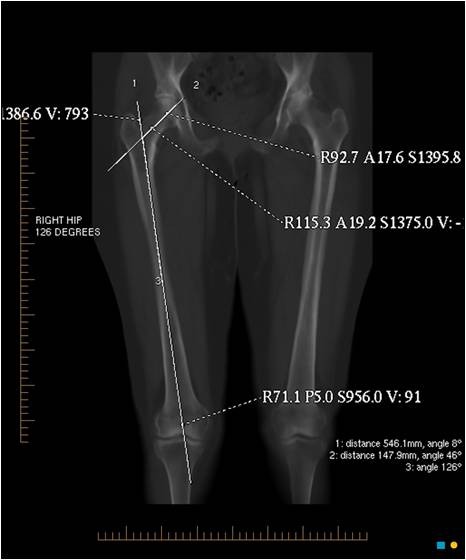
**The femoral neck angle determined by plotting a line along the axis of the femoral neck and a second line along the long axis of the femur**.

The femoral neck angle was determined by plotting a line along the axis of the femoral neck and a second line along the long axis of the femur. The transection of these lines gave the femoral neck angle (Figure 4).

**Figure 5:**
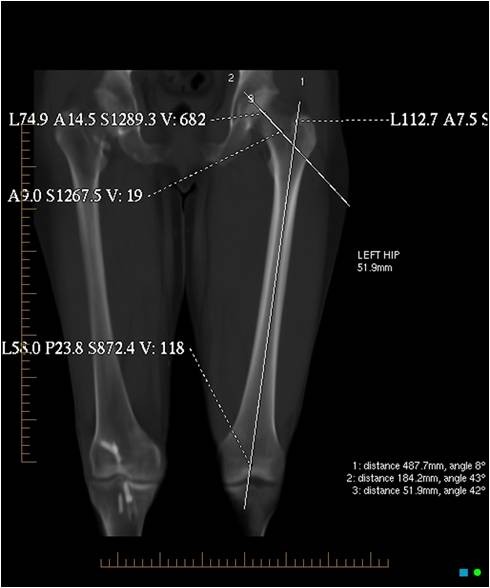
**The femoral offset determined as the distance from the centre of rotation of the femoral head to a line bisecting the long femoral axis**.

The femoral offset was determined as the distance from the centre of rotation of the femoral head to the line bisecting the long femoral axis (Figure 5) [19].

Data were statistically analyzed using the Friedman non-parametric test allowing for two-way analysis of variance, after Kolmogorov-Smirov analysis. Data were also analyzed using the Wilcoxon Signed Ranks Test. A 2-tailed comparison was made of patients' left and right hip data using Spearman's correlation tests.

## Results

The analyzed results demonstrated no sex differences in femoral torsion/anteversion, femoral neck angle and acetabular inclination (Table 1). Males had a mean femoral torsion/anteversion of 8 degrees (range -5 to 26 degrees), a mean femoral neck angle of 129 degrees (range 119 to 138 degrees) and a mean acetabular inclination of 55 degrees (range 40 to 86 degrees). Females had a mean femoral torsion/anteversion of 9 degrees (range -2 to 31 degrees), a mean femoral neck angle of 128 degrees (range 121 to 138) and a mean acetabular inclination of 57 degrees (range 44 to 80 degrees).

**Table 1:** Demonstrates no differences in femoral torsion or femoral neck angle between the sexes

	Sex	N	Mean Rank	Sum of Ranks	Significance
Femoral Torsion/deg	Male	61	45.04	2522.00	Z=-0.865
	Female	39	49.97	1849.00	p = 0.387
	Total	100			No significance

Femoral Neck Angle/deg	Male	61	137.43	2656.00	Z=-0.189
	Female	39	136.35	1715.00	p = 0.850
	Total	100			No significance

Females had a significantly greater acetabular version of 23 degrees (range 10 to 53) compared with 18 degrees in males (range 7 to 46 degrees (p = 0.02) and males had a significantly greater femoral offset of 55 mm (range 42 to 68 mm) compared with 48 mm (range 37 to 57 mm) in females (p = 0.00) (Table 2). There were no significant differences between measurements taken from each patient's right and left sides (Tables 3 and 4). There was no significant difference in the proportion of acetabular or femoral dysplasia between the sexes.

**Table 2:** Demonstrates differences in the acetabular version and femoral offset between the sexes

	Sex	N	Mean	Std. Deviation	Std. Error Mean	Significance t-test and p	Significance
Acetabular Version/deg	Male	61	17.2	9.28	1.240	t = 2.320	*
	Female	39	22.59	10.33	1.698	**p = 0.023**	

Acetabular Inclination/deg	Male	61	54.89	7.23	0.967	t = -1.115	None
	Female	39	56.65	7.73	1.270	p = 0.260	

Femoral Offset/mm	Male	61	55.36	5.82	0.778	t = 6.000	**
	Female	39	48.17	5.19	0.853	**p = 0.000**	

* Females have greater acetabular version

** Males have a greater femoral offset

**Table 3:** Demonstrates no significant differences between measurements taken from each patient's right and left hips

	Paired Samples Statistics	Mean	N	Std. Deviation	Std. Error Mean	Significance	Significance
Pair 1	Acetabular Version/deg	70.08	100	10.11	1.69	t = 1.546	No
	Acetabular Version/deg	68.72	100	11.01	1.84	p = 0.131	
Pair 2	Acetabular Inclination/deg	56.50	100	7.09	1.18	t = 0.199	No
	Acetabular Inclination/deg	56.31	100	8.85	1.47	p = 0.844	
Pair 3	Femoral Offset/mm	52.14	100	6.79	1.13	t = 0.799	No
	Femoral Offset/mm	51.66	100	6.71	1.12	p = 0.430	

**Table 4:** Demonstrates no significant differences between measurements taken from each patient's right and left hips

**Test Statistics**^**c**^		
	**Femoral Torsion/deg - Femoral Torsion/deg**	**Femoral Neck Angle/deg - Femoral Neck Angle/deg**

Z	-1.40^a^	-0.995^b^
Asymp. Sig. (2-tailed)	0.161	0.320

a. Based on negative ranks.

b. Based on positive ranks.

c. Wilcoxon Signed Ranks Test

There was no significant intraobserver (P = 0.72, Wilcoxon rank test) or interobserver error (P = 0.19, Friedman test).

## Discussion

This study has demonstrated significant differences in the femoral offset and acetabular version between males and females, with no significant differences between the other pelvic morphological parameters. Males had a greater femoral offset, and females had greater acetabular version. These results differ from the literature where females have been reported as having greater femoral neck anteversion and lower femoral neck-shaft angles [12-17]. This discrepancy may be the result of the racial uniformity seen in our patient group; certainly racial variations in hip morphology have been previously reported [20,21]. A study of 50 white women and 50 black women found that hip axis length and the neck width were significantly longer in the white women, while neck/shaft angles were not statistically different in the two groups [20].

This study also found no significant differences in morphology between patients' right and left hips, which is also contrary to findings from another study which concluded that side-specific prostheses should be manufactured [22].

While adjustments to anatomically restore the femoral offset and femoral version are possible using modular neck prostheses in total hip arthroplasty [12], we believe that attention should also be paid to the natural acetabular version. We emphasise the importance of pre-operative CT scanning in accurately determining these parameters, to allow for optimum component positioning, particular in the context of hip resurfacing arthroplasty.

## Abbreviations

CT: Computed Tomography

## Consent

Written informed consent was obtained from all patients for their data inclusion in this and other research at our Institution. Copies of these consent forms are available for review by the Editor-in-Chief of this journal

## Competing interests

The authors declare that they have no competing interests.

## Authors' contributions

All the patients underwent arthroplasty surgery by RO. HA wrote the manuscript. HA, SZ and CWO defined and measured the radiological parameters. KJ assisted with the literature review and manuscript preparation. All authors have read and approved the final manuscript.
